# *Clostridioides difficile* infection in trials of short versus long duration of antimicrobials

**DOI:** 10.1017/ash.2024.45

**Published:** 2024-05-06

**Authors:** Dimitri M. Drekonja, Peyton Smith

**Affiliations:** 1 Minneapolis Veterans Affairs Health Care System, University of Minnesota Medical School, Minneapolis, MN, USA; 2 Department of Medicine, University of Minnesota, Minneapolis, MN, USA; 3 Whitman College, Walla Walla, WA, USA

## Introduction

*Clostridioides difficile* infection (CDI) is a significant adverse effect of antimicrobial use, causing significant harms and costs. These harms include an estimated 453,000 cases/year, 83,000 episodes of recurrence, 27,300 deaths,^1^ and annual costs in the United States of 1.3–3.4 billion dollars.^2^ These impacts have led to *C. difficile* being categorized as an “urgent threat” in the US Centers for Disease Control and Prevention’s 2019 Antimicrobial Resistance Threat Report.^3^

Antimicrobial use is the cause of most CDI episodes, and preventing CDI is one of the motivations for reducing unnecessary antimicrobial use. Whether antimicrobial duration influences CDI risk is unclear; it may be that antimicrobial exposure is binary (exposed or unexposed), or that increasing antimicrobial duration impacts risk of CDI. With incidence of CDI after antimicrobial use typically close to 1%, individual trials of shorter versus longer durations of antimicrobial therapy for different infections have too few cases of CDI to answer this question. However, as the number of trials has increased, pooled data allows evaluation of the impact of antimicrobial duration on the risk of developing CDI. We sought to evaluate the impact of antimicrobial duration on the development of CDI by conducting a systematic review and metanalysis of randomized controlled trials (RCTs) comparing durations of antimicrobial therapy and subsequent CDI.

## Methods

We searched for trials of antimicrobial treatment duration that included CDI as an outcome, using PubMed searches followed by review of citations in identified trials. Inclusion criteria were: (1) RCT comparing two treatment durations of antimicrobial agents, (2) shorter duration being ≥3 days, (3) difference between shorter and longer duration being ≥3 days, and (4) CDI was reported as an outcome stratified by treatment duration. Trials in a language other than English were excluded because we lacked resources for translation. Cochrane RevMan version 7.1.1 was used to compare the number of CDI cases among participants receiving shorter versus longer duration, calculate a fixed Mantel-Haenszel odds ratio (OR), and an I^2^ statistic for heterogeneity. Literature review and data abstraction were conducted by one author (PS) and verified by another (DD), and discrepancies addressed by discussion and agreement. The literature search was conducted between 6/2022 and 9/2022.

## Results

We identified 115 potential studies, with 27 removed after title or abstract review, and 88 undergoing full-text review. Of these, 76 were excluded (38 did not report diarrhea or CDI as an outcome, 37 reported diarrhea but not CDI, and one reported CDI but not stratified by duration), leaving 12 studies for analysis (Table 1). Among included studies, the median number of subjects was 291 (range, 31–666), with median shorter duration being seven (interquartile range, 6–28) and median longer duration being 14 (interquartile range, 10–48). There were 32 CDI cases among the 3,882 participants (0.82%), with 20 occurring in participants receiving shorter duration, versus 12 in those receiving longer duration (OR 1.62, 95% CI 0.81–3.25; I^2^ = 0%). Method of CDI testing was never specified.

**Table 1.** Randomized controlled trials of shorter versus longer treatment duration reporting rates of *Clostridioides difficile* infection by treatment arm

Author, date	• Infection • Antimicrobial treatment	Length of shorter duration (days)	Length of longer duration (days)	Participants (N)	Total CDI cases	CDI cases in shorter duration	CDI cases in longer duration
Moran, 2014^4^	• Acute bacterial skin and skin-suture infections • Tedizolid and linezolid	6	10	666	0	0	0
Bernard, 2014^5^	• Pyogenic vertebral osteomyelitis • Therapy per clinician	72	144	351	4	2	2
Sawyer, 2015^6^	• Intraabdominal infection • Therapy per clinician	4	8	518	8	5	3
Yahav, 2018^7^	• Gram-negative bacteremia • Therapy per clinician	7	14	604	4	3	1
Daneman, 2018^8^	• Bacteremia • Therapy per clinician	7	14	115	4	3	1
Benkabouche, 2019^9^	• Orthopedic implant infections • Multiple specified options	28	42	123	0	0	0
Gjika, 2019^10^	• Native joint infection • Therapy per clinician	24	48	154	0	0	0
Ahmed, 2020^11^	• Intraabdominal infection • Therapy per clinician	10	28	31	0	0	0
von Dach, 2020^12^	• Gram-negative bacteremia • Therapy per clinician	7	14	333	6	2	4
Dinh, 2021^13^	• Community-acquired pneumonia • Amoxicillin/clavulanate	3	8	310	1	1	0
Bernard, 2021^14^	• Prosthetic Joint infection • Therapy per clinician	72	144	404	3	2	1
Drekonja, 2021^15^	• Urinary tract infection • TMP/sulfa or ciprofloxacin	7	14	272	2	2	0

Abbreviations. CDI, *Clostridioides difficile* infection; TMP/sulfa: trimethoprim/sulfamethoxazole.

## Discussion

Among RCTs of treatment duration, CDI was not reported as an outcome in more than 70 studies, limiting our ability to assess the effect of treatment duration on CDI. Among the 12 studies that did report CDI outcomes stratified by treatment, CDI was a rare event (<1%), and not significantly associated with longer or shorter durations of antimicrobials. Although there is currently insufficient evidence to state that longer durations of antimicrobials confer an increased risk of CDI, there are other factors that contribute to the decision of how long to treat an infection (efficacy, convenience, cost, impact on antimicrobial resistance, drug-drug interactions, and other adverse drug effects). Limitations include no information on type or timing of CDI testing or the clinical circumstances. Type and duration of therapy varied by study (Table 1), and two studies contributed 44% of all cases. None of the included trials had sufficient power to detect a difference in CDI rates; one pilot study reported that an ongoing trial will have 85% power to detect a reduction in CDI to 3% from 5%.^8^ In addition to being adequately powered, future trials should collect data on CDI with standardized methods and criteria for testing.
